# Persistent Polyclonal B-Cell Lymphocytosis with Binucleated Lymphocytes

**DOI:** 10.4274/tjh.galenos.2021.2021.0061

**Published:** 2021-06-01

**Authors:** Berrin Balık Aydın, Yaşa Gül Mutlu, Ömür Gökmen Sevindik

**Affiliations:** 1 Medipol İstanbul University, Department of Hematology, İstanbul, Turkey

**Keywords:** Lymphocytes, B-cell neoplasms, Lymphoid cell neoplasms, Other lymphoproliferative disorders

## To the Editor,

A 46-year-old female was admitted to our clinic suffering from long-standing leukocytosis. She was evaluated at another hospital regarding this lymphocytic leukocytosis (absolute lymphocyte count: 10770/µL) with no final diagnosis despite further work-up including bone marrow sampling.

**Figure 1:**
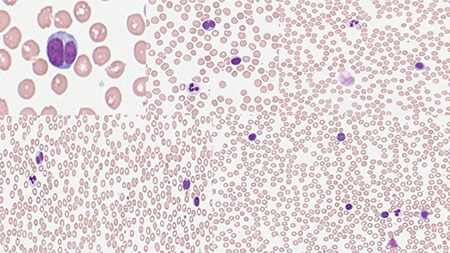
Abundant binucleated lymphocytes with some ghost cells. May-Grunwald-Giemsa staining, 100^x^.

She was a heavy smoker for at least 50 pack-years. We wanted to reassess the underlying disease and ordered a new complete blood count and a peripheral blood smear (Figure 1). We noticed the abundance of binucleated lymphocytes in the peripheral smear.

**Figure 2:**
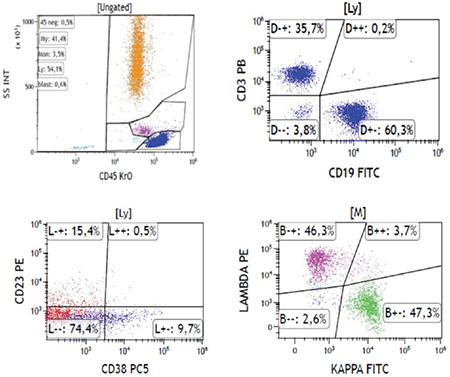
Immunophenotypic features of non-clonal B lymphocytes.

Persistent polyclonal B-cell lymphocytosis (PPBL) does not have a distinctive phenotype. Flow cytometry is performed for the exclusion of a clonal B lymphoid disorder (Figure 2). The patient was diagnosed with PPBL according to the further work-up and was informed about the benign nature of the disease.

PPBL is an infrequent benign disease first described in 1982, characterized by the abundance of binucleated lymphocytes [1]. The immunophenotype of PPBL shows an expansion of B-cells that usually express CD19, CD20, CD22, CD27, and CD79b and are commonly negative for CD5, CD10, CD23, and CD38, with a normal kappa/lambda light chain ratio [2,3]. PPBL often shows an indolent, stable course over many years or slight progress with continued smoking, so the diagnosis of PPBL is crucial in order to avoid unnecessary procedures and therapeutic measures.
